# The Impact of *Lactobacillus plantarum* WCFS1 Teichoic Acid D-Alanylation on the Generation of Effector and Regulatory T-cells in Healthy Mice

**DOI:** 10.1371/journal.pone.0063099

**Published:** 2013-04-30

**Authors:** Maaike J. Smelt, Bart J. de Haan, Peter A. Bron, Iris van Swam, Marjolein Meijerink, Jerry M. Wells, Michiel Kleerebezem, Marijke M. Faas, Paul de Vos

**Affiliations:** 1 Top Institute Food and Nutrition, Wageningen, The Netherlands; 2 University Medical Center Groningen and University of Groningen, dept. Pathology and Medical Biology, Groningen, The Netherlands; 3 NIZO Food Research, Ede, The Netherlands; 4 Wageningen University, dept. Host-Microbe Interactomics Group, Wageningen, The Netherlands; 5 Kluyver Centre for Fermentation and Genomics, Delft, The Netherlands; Virginia Tech, United States of America

## Abstract

To date it remains unclear how probiotics affect the immune system. Bacterial envelope components may play an essential role, as these are the first to establish bacterial-host cell interactions. Teichoic acids (TAs), and especially lipoteichoic acids, are the most pro-inflammatory components of the gram-positive bacterial envelope. This effect is dependent on D-alanyl substitution of the TA backbone and interactions with TLR2 on host cells. Although the pro-inflammatory properties of TAs have been established *in vitro*, it remains unclear how TAs affect immunomodulation *in vivo*. In this study, we investigated the role of TA D-alanylation on *L. plantarum–*induced intestinal and systemic immunomodulation *in vivo*. For this, we compared the effect of *L. plantarum* WCFS1 and its TA D-Alanylation negative derivative (*dltX-D*) on the distribution of dendritic cell and T cell populations and responses in healthy mice. We demonstrated that the majority of the *L. plantarum-*induced *in vivo* immunomodulatory effects were dependent on D-alanylation (D-Ala), as some *L. plantarum* WCFS1-induced immune changes were not observed in the *dltX-D-*treated group and some were only observed after treatment with *dltX-D*. Strikingly, not only pro-inflammatory immune responses were abolished in the absence of D-Ala substitution, but also anti-inflammatory responses, such as the *L. plantarum*-induced generation of regulatory T cells in the spleen. With this study we provide insight in host-microbe interactions, by demonstrating the involvement of D-alanylation of TAs on the bacterial membrane in intestinal and systemic immunomodulation in healthy mice.

## Introduction

The precise mechanisms by which different probiotics impact the mammalian immune system have yet to be discovered. It is likely that extracellular bacterial factors play a pivotal role, as these molecules establish the first interactions between the bacteria and host cells [1]–[3]. For the lactic acid bacterium (LAB) *Lactobaccilus plantarum* WCFS1, a single colony isolate of the strain NCIM8826 [4], it has been demonstrated that its immunomodulatory properties *in vitro* depend on the presence of specific cell-envelope molecules [5], [6]. Even subtle differences in the composition of these molecules can induce large differences in the host cell immune response [6]–[8]. The exact role of these molecules and the type of host response they generate *in vivo* remains to be identified.

Teichoic acids are part of the gram-positive bacterial envelope and recognized as immunomodulating effector molecules [9]–[13]. The majority of LAB produce two types of teichoic acids (TAs); wall teichoic acid (WTA) and lipoteichoic acid (LTA). WTA is covalently anchored to the Mur*N*AC residue of peptidoglycan via a phosphodiester bond. LTA is attached in the cytoplasmic membrane through a glycolipid anchor [14], [15]. While the biosynthesis of LTA is conserved among LAB, some LAB, such as *L. rhamnosus, L. casei, L. fermentum,* and *L. reuteri,* are unable to produce WTA [15].

Especially LTA has been recognized as one of the most immunomodulating cell wall components in gram-positive bacteria [9]–[13]. Although the potency differs between bacterial strains [10], it has been demonstrated that LTA purified from *L. plantarum* NCIMB8826 can induce a potent pro-inflammatory response in immune cells *in vitro* [11], [12]. This response was dependent on D-alanyl substitution of the LTA backbone, its glycolipid anchor [16], [17], and interaction with the pattern recognition receptor Toll-like receptor-2 (TLR-2) on host immune cells [11]. Indeed, absence of TA D-alanylation (D-Ala) shifted the capacity of *L. plantarum* NCIMB8826 and purified LTA to modulate immune responses *in vitro* towards a more anti-inflammatory cytokine profile [11]. Although both LTA and WTA lack D-Ala in this study, the effects can be attributed to LTA, as WTA lacks the immunogenic glycolipid anchor [17]. Moreover, purified *L. plantarum* WTA is unable to activate TLR-2 and to provoke a cytokine response in immune cells *in vitro* [8]. *In vivo*, it has been demonstrated that absence of TA D-Ala improves the protective effect of *L. plantarum* NCIMB8826 in a mouse colitis model, as compared to the wild-type strain [11]. Similar results have been obtained with an *L. rhamnosus* GG mutant that is deficient in D-Ala substitution of LTA [18] and an *L. acidophilus* NCFM mutant that is unable to synthesize LTA [19]–[21]. The latter mutant was able to normalize pathogenic innate and adaptive immune responses, resulting in regression of established colonic polyps in a mouse model [22].

These results support the general hypothesis that LTAs predominantly generate pro-inflammatory immune responses [9]–[13] and that the absence of functional LTAs in the bacterial membrane improve the bacterial anti-inflammatory capacity [11], [18]–[22]. Although several specific LTA-induced pro-inflammatory immune effects have been demonstrated *in vivo* [21], it remains unclear how LTAs influence immune cell populations *in vivo*. In the present study, we aimed to investigate the effects of *L. plantarum* LTA on the distribution of adaptive immune cell populations in healthy animals *in vivo*. For this, we compared the effects the probiotic strain *L. plantarum* WCFS1 [23] and its D-Ala negative derivative (*dltX-D*) [8] on the distribution of intestinal and systemic T cell and dendritic cell (DC) populations in healthy mice. The bacteria were administrated orally for 5 days, which is the period to develop an adaptive immune response [24], [25]. Moreover, since *L. plantarum* poorly colonizes the gastrointestinal tract [26], daily inoculation ensured the presence of the bacteria in the gastrointestinal tracts of the mice during the complete course of the experiment. We demonstrate that the distribution of not only pro-, but also anti-inflammatory T cell and DC populations is dependent on the functionality of the *dltX-D*-encoded system that D-alanylates TAs in the *L. plantarum* WCFS1 cell envelope.

## Materials and Methods

### Bacterial Growth Conditions

Wild-type *L. plantarum* WCFS1 (referred to as *WT*) [4] and *ΔdltX-D,* a WCFS1 derivative that was confirmed to be defective in D-alanylation of TA (NZ3539Cm; referred to as *dltX-D*) [8], were cultured at 37°C in Man, Rogosa, and Sharpe (MRS) broth. An overnight culture was diluted 1∶1000 and cultured overnight, so that the bacteria were in the stationary phase. The optical density at 600 nm was measured and the number of colony forming units (CFU) was calculated based on the confirmed correlation that an OD_600_-value of 1 corresponds to 1–2×10^9^ CFU/mL for each strain used.

### *In vitro* Culture and Stimulation of Murine HEK293 mTLR Reporter Cells

5×10^5^ cells/cm^2^ human embryonic kidney (HEK)293 cells harbouring murine TLR2/1 or TLR2/6 combined with pNIFTY, a NKκB luciferase reporter construct (Invivogen, Toulouse, France), were plated in 96-wells plates and cultured overnight at 37°C 5% CO_2_. Subsequently the cells were incubated in triplicate with WT-*L. plantarum* or *L. plantarum dltX-D* at a concentration of 15 colony forming units (CFU)/HEK293 cell (*N = 6*). Culture medium alone was used as a negative control and TLR2 signaling was always confirmed using the TLR2 ligand Pam3CSK4 (5 µg/mL) (data not shown). NKκB activation was measured using the Bright-glo luciferase assay(promega, Benelux BV, Leiden, The Netherlands).

### *In vitro* Culture and Stimulation of Murine Dendritic Cells

Bone marrow cells were isolated and cultured as described by Lutz *et al* [27], with minor modifications. Briefly, femora and tibiae from female 6 weeks old Balb/c mice (Charles River Breeding Laboratories, Protagem MI), were removed and stripped of muscles and tendons. After soaking the bones in 70% ethanol and rinsing in PBS, bones were carefully crushed with a mortar to release the bone marrow cells. Cells were filtered using Steriflip filtration and washed with RPMI medium. Bone marrow cells (2–4×10^7^) were seeded into Petri dishes in 10 ml RPMI 1640 Glutamax (Sigma–Aldrich, St. Louis, MO, USA) containing 10% (v/v) heat-inactivated fetal calf serum supplemented with penicillin (100 U/ml), streptomycin (100 µg/ml), 50 µm β-mercaptoethanol and 20 ng/ml murine GM-CSF (R&D systems). The cells were incubated for 8 days at 37°C in 5% CO_2_ humidified atmosphere. On day 3, 10 ml was removed and replaced with complete medium. On day 5, 5 ml fresh medium was added. On day 7, immature dendritic cells were collected and seeded in a 24 wells plate at 5×10^5^ cells/well. On day 8, the cells were either left unstimulated or stimulated with *L. plantarum* WCFS1 or *L. plantarum dltX-D* (1∶10 cell to bacteria ratio) (*N = 4*), or LPS (1 µg/mL). After 24 hours the concentration of IL10 and TNFα was determined in the culture supernatants using cytometric bead array (BD Biosciences).

### *In vivo* Probiotic Treatment and Distribution of Immune Cell Populations

Wild-type male Balb/c mice were purchased from Harlan (Harlan, Horst, The Netherlands). The animals were fed standard chow and water *ad libitum*. All animal experiments were performed after receiving approval of the institutional Animal Care Committee of the Groningen University. The size of the experimental groups (*N = 6*) was based on mandatory power calculations. All animals received animal care in compliance with the Dutch law on Experimental Animal Care.

The mice received either sterile MRS broth or 1–2×10^8^ CFU bacteria (*WT* or *dltX-D*) in 200 µL MRS broth via intragastric gavage, daily for five consecutive days. This bacterial load was chosen based on the protective effects of a D-Ala negative derivative strain of *L. plantarum* (*dltB*) in a T cell dependent colitis model [11]. On day six, the mice were sacrificed by cervical dislocation, after which the intestine, spleen, and mesenteric lymph nodes were removed for further analysis.

### Isolation of Lamina Propria and Peyer’s Patch Leukocytes

After removal, the intestine was rinsed with ice cold Phosphate Buffered Saline (PBS). Peyer’s Patches (PPs) were removed from the tissue and single cell suspensions of the PPs were made by mechanical disruption of the tissue between two glass slides in 1 mL of ice cold RPMI containing 10% (v/v) heat inactivated fetal calf serum (FCS). Subsequently, a cell strainer was used to remove remaining clumps.

The small and large intestine were cut in small pieces and rinsed three times in ice cold PBS. Epithelial cells were removed by incubation of the tissue in PBS containing 10% (v/v) FCS, 1 mM Sodium Pyruvate, 10mM Ethylenediaminetetraacetic (EDTA) and 20 mM 4-(2-hydroxyethyl)-1-piperazine-ethanesulfonic acid (HEPES) (pH 7.4) for 30 minutes at 37°C, shaking. The lamina propria was removed by incubation of the tissue in RPMI 1640 medium, containing 10% (v/v) FCS, 1.5 mg/mL Collagenase D (Sigma Aldrich), and 10 mg/mL DNAse I (Sigma Aldrich), for 60 minutes at 37°C, shaking. The reaction was terminated by adding EDTA to a final concentration of 10 mM. The cell suspensions were washed in ice cold PBS and a cell strainer was used to remove remaining clumps.

To enrich lymphocytes and to remove dead cells, the PP and lamina propria cell mixtures were loaded on a percoll gradient of 55%, 45%, 35%, and 20% (GE Healthcare, Eindhoven, the Netherlands) and centrifuged for 30 minutes at 800×*g,* at room temperature (RT). The interface was washed in ice cold PBS, counted and used for staining. After density gradient centrifugation, more than 90% of the cells were vital, which was confirmed by propidium iodide staining.

### Spleen and MLN Cell Isolation and Stimulation

Spleen and MLN single cell suspensions were made by mechanical disruption of the tissue between two glass slides in 1 mL of ice cold RPMI containing 10% (v/v) FCS. Subsequently a cell strainer was used to remove remaining clumps. The cells were washed, counted, and used for staining.

Part of the cells of the spleen and MLN were *ex vivo* restimulated, the rest was left unstimulated. 7×10^6^ cells from the spleen and MLN were stimulated in RPMI 10% FCS containing 40 nM Phorbol 12-myristate 13-acetate (PMA) (Sigma Aldrich) and 2 nM calcium ionophore (Ca^2+^) (Sigma Aldrich). Monensin (3 µM) (Sigma Aldrich) was added to allow cytokine accumulation in the cellular cytoplasm. Cells were stimulated for four hours at 37°C, after which they were washed in ice cold PBS containing 2% (v/v) FCS (FACS buffer), and used for staining.

To enrich dendritic cells and to remove dead cells, the spleen and MLN cell mixtures were loaded on ‘1-step Monocyte’ (Accurate Chemical and Scientific Corporation, Westbury, NY) with a density of 1.068±0.001 g/ml, and centrifuged for 30 minutes at 300×*g* at 4°C. The interface was washed twice in ice-cold FACS buffer and used for staining. After density gradient centrifugation, more than 90% of the cells were vital, which was confirmed by propidium iodide staining.

### Cell Staining

T cell stainings were performed on non-stimulated splenic, MLN, PP, and lamina propria cell suspensions. DC stainings were performed on non-stimulated, DC-enriched splenic, MLN, PP, and lamina propria cell suspensions. Stainings for intracellular cytokines were performed on PMA/Ca^2+^ stimulated splenic and MLN cell suspensions. The T cell cocktail contained monoclonal antibodies directed against CD3, CD4, CD8, CD25, CD69, FoxP3, or appropriate isotype controls (Table 1). The DC cocktail contained monoclonal antibodies directed against CD11c, MHC II, CD19, CD80, CD103, or appropriate isotype controls (Table 1). The effector T cell cocktail contained monoclonal antibodies directed against CD3, CD4, CD8, IFNγ, IL5, IL10, IL17, or appropriate isotype controls (Table 1).

**Table 1:** Antibodies.

*Specificity*	*Clone Name*	*Fluorochrome*	*Dilution*	*Supplier*
CD3	17A2	Pacific Blue	200x	BioLegend
CD4	RM4–5	PerCP	200x	BioLegend
CD8	53–6.7	Alexa700	50x	BioLegend
CD25	3C7	APC	100x	BioLegend
CD69	H1.2F3	PE	200x	BioLegend
FoxP3	FJK-16S	FITC	100x	eBioscience
IFNγ	XMG1.2	APC	100x	BioLegend
IL5	TRFK5	PE	25x	BioLegend
IL10	JES5-16E3	PE	25x	BioLegend
IL17a	TC11-18H10.1	APC	25x	BioLegend
Rat IgG2b	N/A	APC	100x	BioLegend
Hamster IgG	N/A	PE	200x	BioLegend
Rat IgG2a	N/A	FITC	100x	eBioscience
Rat IgG1	N/A	APC	25x or 100x	BioLegend
Rat IgG1	N/A	PE	25x	BioLegend
Rat IgG2b	N/A	PE	25x	BioLegend
CD11c	N418	APC	25x	BD Biosciences
MHC II	2G9	Biotin+streptavidin PerCP	150x	BD Biosciences
CD19	6D5	PE-Cy7	100x	BioLegend
CD80	16-10A1	PE	50x	BioLegend
CD86	PO3	Alexa700	50x	BioLegend
CD103	2E7	Pacific Blue	25x	BioLegend
Hamster IgG	N/A	PE	50x	BioLegend
Rat IgG2b	N/A	Alexa700	50x	BioLegend
Hamster IgG	N/A	Pacific Blue	25x	BioLegend

In short, 1×10^6^ cells were incubated in FACS buffer containing 10% (v/v) normal mouse serum for 30 minutes to prevent non-specific antibody staining. Subsequently, the cells were incubated with a cocktail of primary antibodies for 30 minutes. The cells were fixed in FACS Lysing solution (BD Biosciences) for 30 minutes, in the dark. The tubes for intracellular cytokine staining were subsequently washed twice in 1× permeabilisation buffer (eBioscience) and incubated with the intracellular antibodies cocktails containing 2% (v/v) normal rat serum in permeabilisation buffer for 30 minutes in the dark. The whole procedure was performed on ice.

### Flow Cytometry

During flow cytometry, at least 5×10^5^ cells were analyzed. Flow Cytometry was performed using the LSR II Flow Cytometer system (BD Pharmingen), using FACS Diva software. Analysis was performed using FlowJo 7.6.2 software. Lymphocytes were gated based on the expression of CD3 and CD4 or CD8. The expression of CD25, CD69, FoxP3, and cytokines was determined based on samples stained with the isotype controls. Dendritic cells were gated in the forward side scatter plot, based on size and granularity. CD19^+^ B-cells were excluded from analysis [28]. DCs were defined as MHC II^+^ CD11c^+^ cells. The expression of CD103 and CD80 within this DC population was determined based on samples stained with the isotype controls.

### Statistics

All data are expressed as the mean ± standard error of the mean (SEM). Normal distribution of the data-sets was confirmed by the Kolmogorov-Smirnov test. The Mann Whitney U test was performed to determine changes in TLR2 and mDC cytokine responses *in vitro.* The two-sided Students t-test was used to determine changes in immune cell populations after probiotic treatment *in vivo.* P-values <0.05 (*) were considered statistically significant. A statistical trend was defined as 0.05<P-value <0.1, which is only described in the text and not depicted in the graphs.

## Results

### Absence of D-Ala Substitution Reduces Murine TLR2 Signaling and Enhances the Anti-inflammatory Immune Modulatory Capacity of *L. plantarum in vitro*

To gain insight in the role of TAs in probiotic-induced immunomodulation in mice *in vivo,* we first confirmed the altered immunomodulatory property of our mutant strain *dltX-D* as compared to the wild-type strain (*WT*) in a murine-based *in vitro* assay. For this, the potential to induce murine TLR2 signaling as well as dendritic cell (DC) cytokine responses were evaluated *in vitro*. Medium and wild-type *L. plantarum* WCFS1 (*WT*) were used as controls.

**Figure 1:**
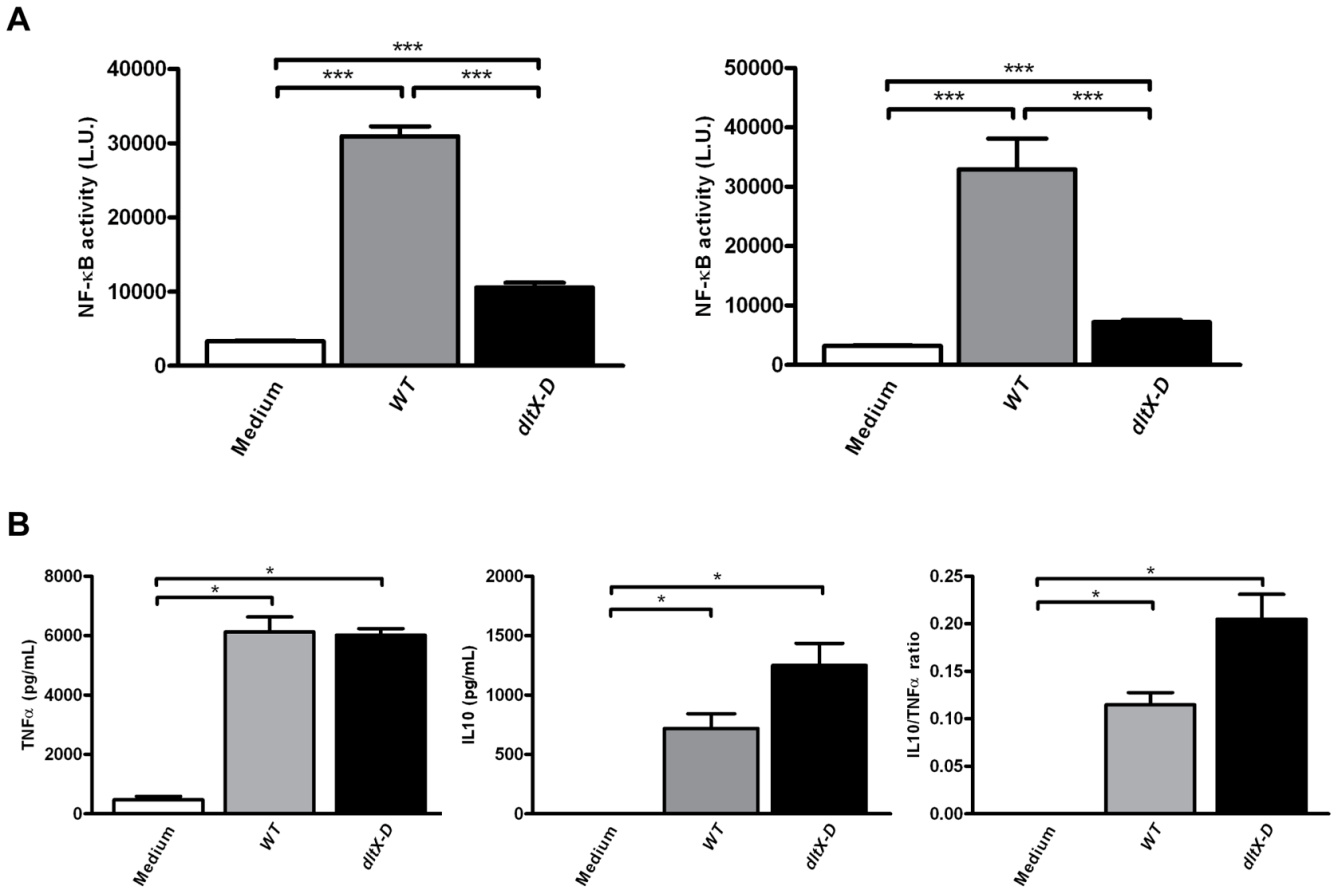
Toll like receptor signaling and mDC cytokine responses *in vitro*. *In vitro* activation of HEK cells containing a murine TLR2/1 or TLR2/6 reporter construct in response WT (grey bars), dltX-D (black bars), or culture medium alone (white bars) (N = 6) (A). Following incubation of murine DCs with medium (white bars), WT (grey bars), or dltX-D (black bars) the release of TNFα or IL10 was determined (N = 4) (B). In addition, the IL10/TNFα ratio was calculated. Results are depicted as the mean ± standard error of the mean (SEM). Statistical significance was calculated using the Mann Whitney U test. *represents P-values <0.05, **represents P-values <0.01, ***represents P-values <0,001.

As expected, *dltX-D* demonstrated significantly decreased TLR2/1 and TLR2/6 activation as compared to *WT* (Figure 1A), although *dltX-D* retained some residual TLR2 signaling capacity, as demonstrated by increased TLR2 signaling as compared to medium stimulated reporter cells (Figure 1A). Further, in murine DCs absence of D-Ala substitution did not affect the *L. plantarum-*induced pro-inflammatory TNFα response (Figure 1B), whereas a trend towards an increased IL10 response and IL10/TNFα ratio (*P = 0.06*) was observed after co-incubation with *dltX-D* as compared to *WT* (Figure 1B). These results demonstrate that absence of *L. plantarum* TA D-Ala affects its pro- and anti-inflammatory immunomodulatory capacity in murine host cells *in vitro*.

### *L. plantarum*-induced Changes in Intestinal DC Frequencies are not Observed in the Absence of TA D-Ala Substitution

**Figure 2:**
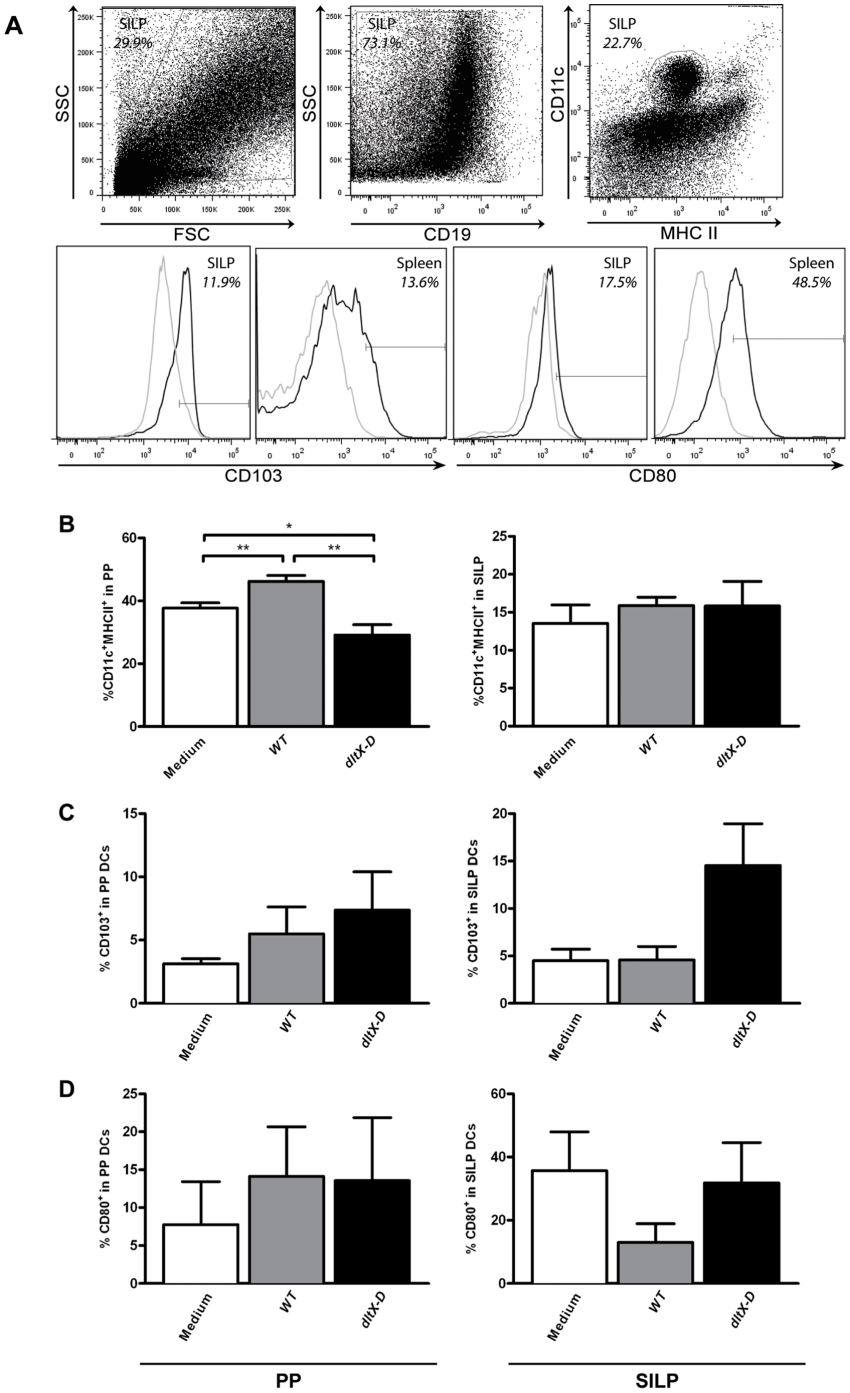
Dendritic cell frequencies and activation in the peyer’s patches and small intestinal lamina propria. Dendritic cells were gated based on size, granularity, and the expression of CD11c and MHC II. CD19^+^ B cells were excluded from analysis. Within the dendritic cell population the frequency of CD103^+^ or CD80^+^ was determined (black lines). The gates were set based on staining with an isotype control (grey lines). Representative FACS plots from the small intestinal lamina propria (SILP) and spleen are depicted (A). Tissue specific staining patterns are demonstrated for both CD103 and CD80, as shown by the FACS plots of SILP and splenic DCs (A). Dendritic cell frequencies in the Peyers Patches and SILP (N = 6) (B) following oral treatment with medium (white bars), WT (grey bars), or dltX-D (black bars). CD103^+^ DC frequencies in the PP and SILP (C). CD80^+^ DC frequencies in the PP and SILP (D). Results are depicted as the mean ± standard error of the mean (SEM). Statistical significance was calculated using the Students t- test. *represents P-values <0.05.

How *L. plantarum* TA D-Ala affects the immune system *in vivo* was investigated in mice. Healthy mice (*N = 6*) received *dltX-D, WT*, or culture medium alone for five consecutive days. First, we measured the changes in the distribution of different DC populations. The intestine was divided in three locations: the Peyer’s Patches, the Small Intestinal Lamina Propria (SILP), and the Large Intestinal Lamina Propria (LILP). On average we retrieved 746.000±7875 cells from the LILP, which was too low to allow for reliable quantification of changes in the DC compartment. DCs were defined as CD11c^+^MHC II^+^ cells (Figure 2A). DCs are depicted as the frequency of CD103^+^ or CD80^+^ cells within the DC population (Figure 2A).

In the intestine, *L. plantarum* TA D-Ala only modestly affected the DC compartment. *DltX-D-*treated mice demonstrated decreased DC frequencies in the PP as compared to *WT-*treated animals and medium treated controls (Figure 2B). This effect was not observed in the SILP (Figure 2B). In the PP, CD103^+^ DC frequencies were not affected by either *WT* or *dltX-D* treatment (Figure 2C), whereas in the SILP a trend towards increased CD103^+^ DC frequencies was observed after *dltX-D* treatment as compared to medium and *WT-*treated mice (*P = 0.05*) (Figure 2C). Both in the PP and SILP, DC activation was not affected by the bacterial treatments (Figure 2D).

### Bacterial Wall Composition Modifies Intestinal (CD25^+^) CD4 and (FoxP3^+^ CD4^+^) T Cell Frequencies

**Figure 3:**
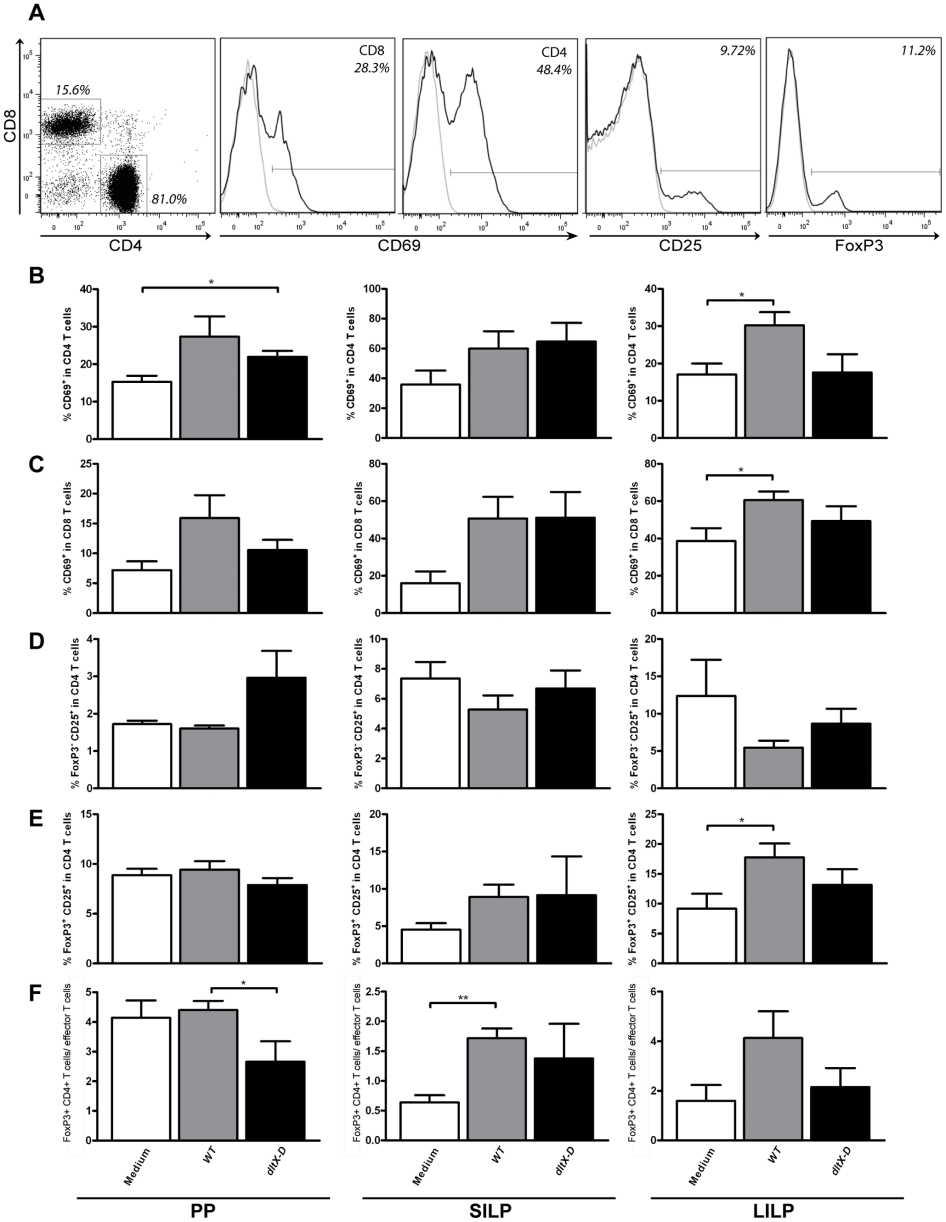
Early activated CD4^+^ and CD8^+^ T cells, effector T cells, and FoxP3^+^ T cells in the Peyer’s patches, small intestinal and large intestinal lamina propria. Within the CD4 or CD8 population the frequency of early-activated cells was determined based on the expression of CD69 (black lines). The gates were set based on staining with an isotype control (grey lines). Effector T cells were gated based on the expression of CD25 (black line) as compared to the isotype control (grey line) within the CD4 T cell population. FoxP3^+^ cells were excluded. FoxP3 were within the CD3^+^CD4^+^ T cell population (black line). The gate was set based on staining with an isotype control (grey line). Representative FACS plots from the small intestinal lamina propria (SILP) are depicted (A). Early activated CD4^+^ T cell frequencies in the PP, SILP, and LILP (N = 6) following oral treatment with medium (white bars), WT (grey bars), or dltX-D (black bars) (B). Early activated CD8^+^ T cell frequencies in the PP, SILP, and LILP (C). Effector T cells in the PP, SILP, and LILP (D). FoxP3^+^ T cell frequencies in the PP, SILP, and LILP (E). Ratio between FoxP3^+^ CD4^+^ T cells and effector T cells in the PP, SILP, and LILP (F). Results are depicted as the mean ± standard error of the mean (SEM). Statistical significance was calculated using the Students t- test. *represents P-values <0.05.

Second, we determined changes in the intestinal T cell compartment. For this, we measured the frequency of early-activated (CD69^+^) CD4 or CD8 T cells, activated (CD25^+^) CD4 effector T cells, and (FoxP3^+^ CD4^+^) T cells (Figure 3A).

In the PP, early-activated CD4 T cell frequencies were increased after both bacterial treatments, as compared to the medium treated controls (Figure 3B). In the *WT*-treated group, this effect was borderline significant (*WT* vs. medium *P = 0.06*) (Figure 3B). In the LILP, early-activated CD4 T cell frequencies were increased after *WT-,* but not *dltX-D* treatment (Figure 3B), whereas in the SILP, early-activated CD4 T cell frequencies were not affected by the bacterial treatments (Figure 3B). In the PP and SILP, the CD8 T cell compartment showed a trend towards increased early-activated CD8 T cells after *WT-*treatment only (*WT* vs. medium *P = 0.08* and *P = 0.06 *resp.) (Figure 3C). In the LILP, the frequency of early-activated CD8 T cells was significantly increased after *WT*- but not after *dltX-D* treatment (*dltX-D* vs. medium *P = 0.33*), as compared to the medium treated controls (Figure 3C).

In addition to the effects on early T cell activation, *L. plantarum* TA D-Ala substitution also had effects on other T cell frequencies in the intestine. In the PP, *WT-*treatment did not affect the distribution of (CD25^+^) CD4 T cells (Figure 3D), or FoxP3^+^ CD4^+^ T cells (Figure 3E), whereas *dltX-D-*treated mice demonstrated a trend towards increased (CD25^+^) CD4 T cell frequencies as compared to the controls (*P = 0.09* vs controls, *P = 0.06* vs *WT*) (Figure 3D). Although the changes in the distribution of PP (CD25^+^) CD4 T cells were not statistically significant, the balance between FoxP3^+^ CD4^+^ and (CD25^+^) CD4 T cells was significantly decreased after *dltX-D-*treatment as compared to *WT-*treatment (Figure 3F). In the SILP and LILP, effector T cell frequencies were not influenced by the bacterial treatments (Figure 3D), but a trend (*P = 0.06*) and significant increase in the frequency of FoxP3^+^ T cells was observed after *WT-,* but not *dltX-D-,* treatment in the SILP and LILP respectively (Figure 3E). Thus, *WT-*treatment increased the balance between FoxP3^+^ and (CD25^+^) CD4 T cells in both the SILP and LILP (*WT* vs. medium *P = 0.07*) as compared to medium treated control animals (Figure 3F). This effect was attenuated in the absence of *L. plantarum* TA D-Ala substitution (Figure 3F).

### D-alanylated TAs Contribute to the *L. plantarum*-induced Increase in Splenic Regulatory DC and T Cell Frequencies

Further, we questioned whether D-Ala substitution of *L. plantarum* TAs also influences immunomodulation in secondary lymphoid organs. For this, we chose the gut-draining lymph nodes (Mesenteric lymph nodes; MLN) and a systemic lymphoid compartment; the spleen. In these compartments we measured the distribution of pro- and anti-inflammatory DC and T cell populations.

**Figure 4:**
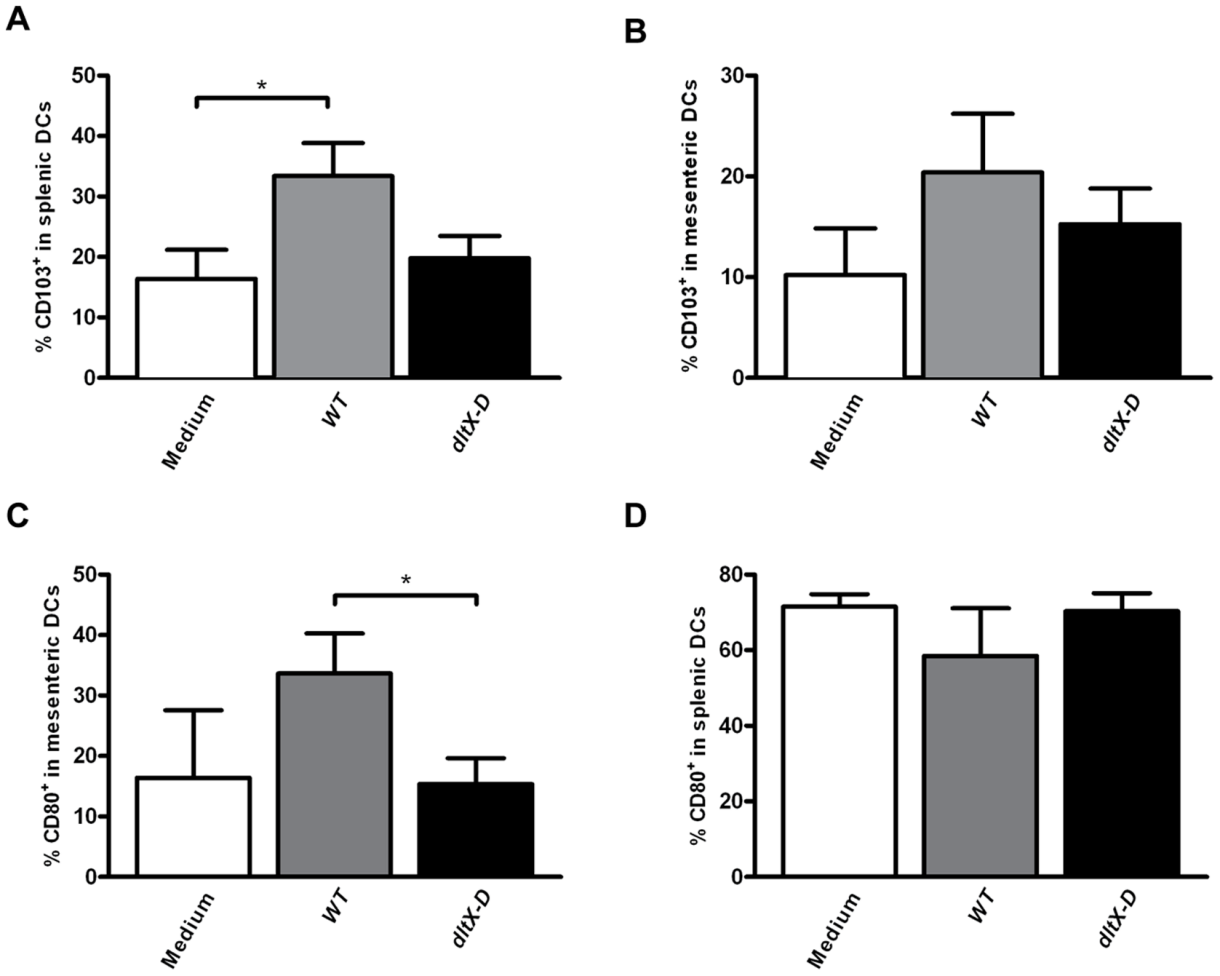
Dendritic cell frequencies and activation in the spleen and mesenteric lymph nodes. Dendritic cells were gated based on the expression of CD11c and MHC II. Within the dendritic cell population the frequency of CD103^+^ or CD80^+^ was determined. CD103^+^ dendritic cell frequencies in the spleen (N = 6) (A) and MLN (B) following oral treatment with medium (white bars), WT (grey bars), or dltX-D (black bars). CD80^+^ DC frequencies in the MLN (N = 6) (C) and spleen (D). Results are depicted as the mean ± standard error of the mean (SEM). Statistical significance was calculated using the Students t- test. *represents P-values <0.05.

D-Ala substitution of TA influenced the translocation of regulatory DCs into systemic immune compartments, as *WT-*treatment increased the frequency of CD103^+^ DCs in the spleen as compared to medium treated controls (Figure 4A), and *dltX-D-*treatment did not (Figure 4A). Although less pronounced, a similar effect was observed in the MLN (*P = 0.09*) (Figure 4B). Similarly, also the trend towards *WT*-induced MLN DC activation (*P = 0.08*) was not observed after *dltX-D* treatment (Figure 4C). Splenic DC activation was not enhanced by any of the bacterial treatments (Figure 4D).

**Figure 5:**
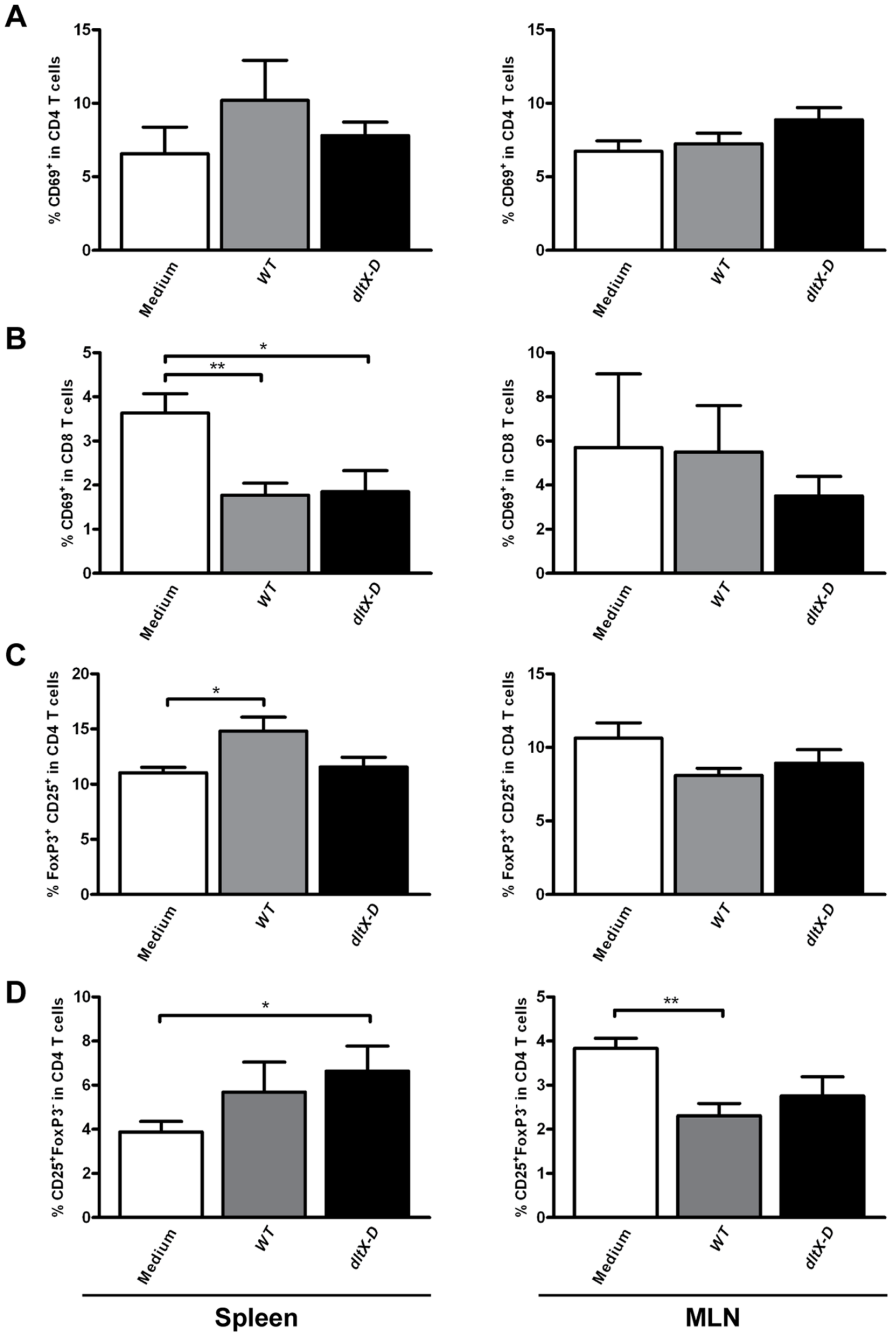
Early activated CD4^+^ and CD8^+^ T cells, effector T cells, and regulatory T cells in the spleen and mesenteric lymph nodes. Within the CD4 or CD8 population the frequency of early activated cells was determined based on the expression of CD69 Effector T cells were gated based on the expression of CD25 within the CD4 T cell population. FoxP3^+^ cells were excluded. Regulatory T cells were gated based on the expression of FoxP3 within the CD3^+^CD4^+^ T cell population Early activated CD4^+^ T cell frequencies in the spleen and MLN (N = 6) following oral treatment with medium (white bars), WT (grey bars), or dltX-D (black bars) (A). Early activated CD8^+^ T cell frequencies in the spleen and MLN (N = 6) (B). Regulatory T cell frequencies in the spleen and MLN (C). Effector T cells in the spleen and MLN (D). Results are depicted as the mean ± standard error of the mean (SEM). Statistical significance was calculated using the Students t- test. *represents P-values <0.05.

In the T cell compartment, the early activation of CD4 T cells in the spleen and MLN (Figure 5A), or CD8 T cells in the MLN (Figure 5B) was not affected by the bacterial treatments. In the spleen, the frequency of early-activated CD8 T cells was significantly decreased after treatment with both *WT* as well as *dltX-D* (Figure 5B). Further, D-Ala substitution was necessary for *L. plantarum* to increase regulatory T cell frequencies in the spleen, as the increase was observed after treatment with *WT,* but not after treatment with *dltX-D* (Figure 5C). Moreover, after *dltX-D-*treatment, the frequency of splenic effector T cells was increased as compared to the medium treated controls (Figure 5D). In the MLN, absence of D-Ala substitution did not affect regulatory T cell frequencies (*dltX-D* vs. medium *P = 0.24*) (Figure 5C), whereas a trend towards decreased regulatory T cell frequencies was observed after *WT-*treatment (*P = 0.06*) (Figure 5C). Both *dltX-D (P = 0.09)* and *WT*-treated mice demonstrated decreased mesenteric effector T cell frequencies (Figure 5D).

### D-alanylation of TAs has a Minor Contribution to *L. plantarum*-induced Modulation of Systemic Polarized T Cell Subsets

**Figure 6:**
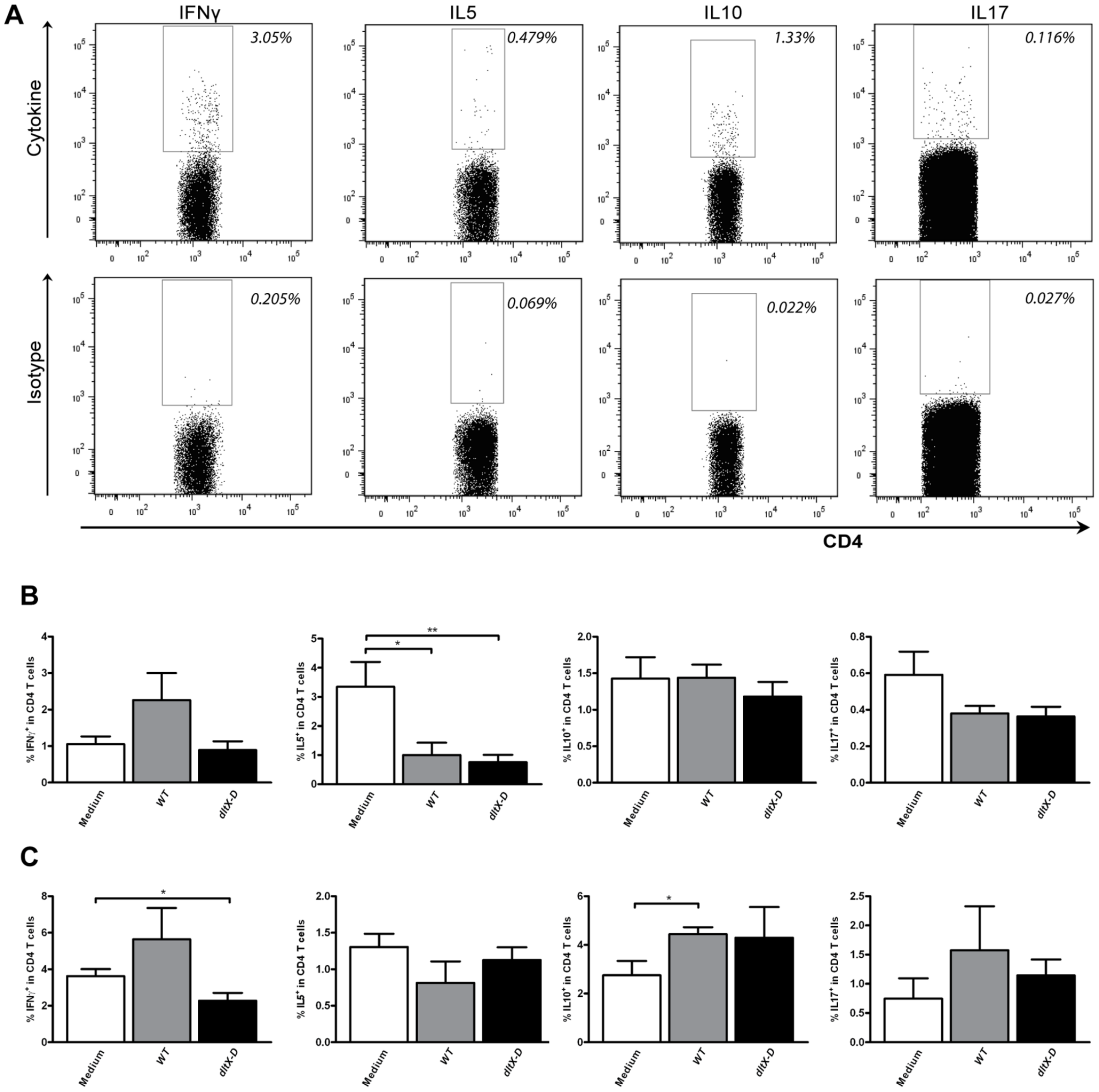
Polarized CD4^+^ T cell frequencies in the spleen and mesenteric lymph nodes. Polarized CD4^+^ T cells were gated based on the expression of IFNγ, IL5, IL10, or IL17 within the CD3^+^CD4^+^ T cell population (top plots). The gate was set based on staining with an isotype control (bottom plots). Representative FACS plots are depicted (A). Polarized CD4^+^ T cell frequencies in the MLN (N = 6) (B) and spleen (C) following oral treatment with medium (white bars), WT (grey bars), or dltX-D (black bars). Polarized CD4^+^ T cell frequencies are depicted as the frequency of IFNγ^+^ cells within CD4^+^ T cells, IL5^+^ cells within CD4^+^ T cells, IL10^+^ cells within CD4^+^ T cells, and IL17^+^ cells within CD4^+^ T cells. Results are depicted as the mean ± standard error of the mean (SEM). Statistical significance was calculated using the Students t- test. *represents P-values <0.05.

In addition to measuring the distribution of DC and T cell subsets, splenic and mesenteric polarized T cell subsets were analyzed. After *ex vivo* PMA/Ca^2+^ stimulation, cellular cytokine responses were determined. IFN-γ was measured as a marker for Th1 cells [29], IL5 was measured as a marker for Th2 cells [29], IL10 was measured as a marker for regulatory T cells [30], and IL17 was measured as a marker for Th17 cells [30], [31]. The frequency of cytokine producing CD4^+^ T cells was determined based on appropriate isotype controls (Figure 6A).

Although some exceptions were observed, most *L. plantarum*-induced effects on splenic and mesenteric polarized T cell subsets were observed in both the *WT-* and *dltX-D-*treated groups. This was demonstrated in the MLN, where *dltX-D* treatment decreased the frequency of Th2 cells in the same fashion as *WT* treatment (Figure 6B). The frequency of Th1, Th17, or IL10-producing T cells was not affected by the treatments (Figure 6B). In the spleen, the frequency of Th1 was decreased after *dltX-D* treatment, which was not observed after *WT* treatment (Figure 6C). The frequency of IL10-producing T helper cells was significantly increased after *WT*, but not *dltX-D* treatment (*dltX-D* vs. medium *P = 0.37*) (Figure 6C).

**Figure 7:**
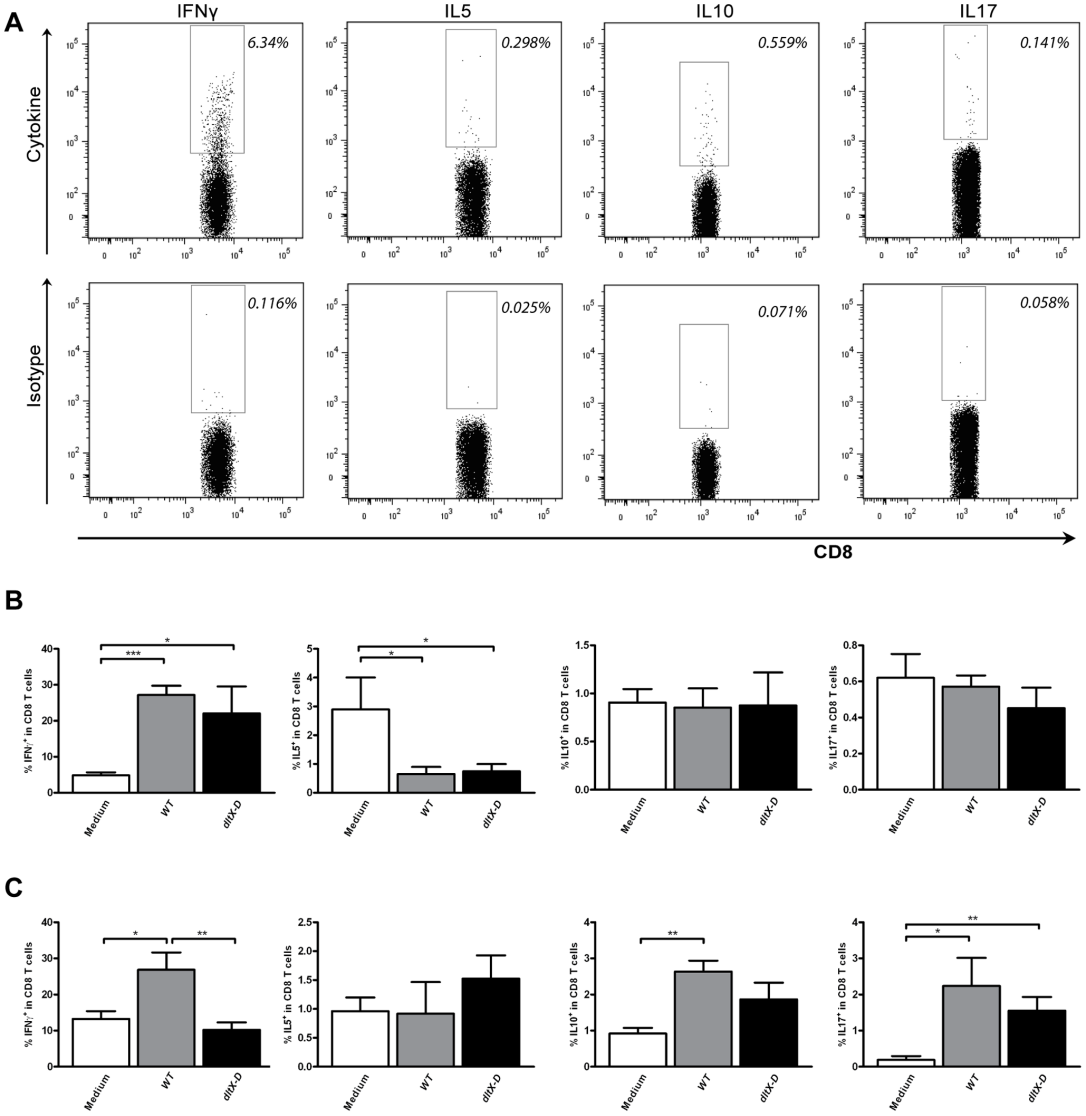
Polarized CD8^+^ T cell frequencies in the spleen and mesenteric lymph nodes. Polarized CD8^+^ T cells were gated based on the expression of IFNγ, IL5, IL10, or IL17 within the CD3^+^CD8^+^ T cell population (top plots). The gate was set based on staining with an isotype control (bottom plots). Representative FACS plots are depicted (A). Polarized CD8^+^ T cell frequencies in the MLN (N = 6) (B) and spleen (C) following oral treatment with medium (white bars), WT (grey bars), or dltX-D (black bars). Polarized CD8^+^ T cell frequencies are depicted as the frequency of IFNγ^+^ cells within CD8^+^ T cells, IL5^+^ cells within CD8^+^ T cells, IL10^+^ cells within CD8^+^ T cells, and IL17^+^ cells within CD8^+^ T cells. Results are depicted as the mean ± standard error of the mean (SEM). Statistical significance was calculated using the Students t- test. *represents P-values <0.05.

Similar cytokine responses can also be observed within CD8^+^ T cells [32]. The frequency of cytokine producing CD8^+^ T cells was determined based on appropriate isotype controls (Figure 7A). In the mesenteric but not the splenic CD8 T cell compartment, *dltX-D* treatment increased the frequency of IFNγ-producing cells in the same fashion as *WT*-treatment (Figure 7B and 7C respectively). Both *dltX-D* and *WT* treatment decreased the frequency of IL5-producing CD8 T cells in the MLN (Figure 7B) and increased the frequency of IL17-producing CD8 T cells in the spleen (Figure 7C). *WT-*treatment increased the frequency of splenic IL10-producing CD8 T cells, which was not observed after treatment with *dltX-D* (*P = 0.21*) (Figure 7C).

## Discussion

Probiotic bacteria are being explored as means to improve health and prevent disease. Both intestinal [33]–[39], as well as peripheral health benefits [40]–[47] have been ascribed to probiotic treatment. One of the proposed mechanisms of action is the modulation of intestinal as well as systemic immunity [24], [48]–[56]. Although the immunomodulatory properties of different probiotic strains have been demonstrated both *in vitro* as well as *in vivo* [24], [48]–[55], the exact mechanisms of action remain obscure, especially in the healthy population. It is still subject of debate where in the intestine probiotic bacteria are sensed and whether direct interactions between the bacteria and the immune system are necessary for immunomodulation *in vivo*.

In the present study we investigated the requirement of TA D-Ala substitution for *L. plantarum* WCFS1-induced modulation of the intestinal and systemic immune system. We first demonstrated that *L. plantarum* TAs signal through murine TLR2 and that the absence of TA D-Ala substitution induces an anti-inflammatory cytokine response in murine DCs in a similar fashion as observed in human immune cells *in vitro* [8], [11]. Further, we demonstrated that 5-day small dose inoculation with *L. plantarum* WCFS1 modulates both intestinal and systemic adaptive immunity. The majority of the *L. plantarum-*induced *in vivo* immunomodulatory effects are dependent on D-Ala substitution, as 15 out of 24 *L. plantarum* WCFS1-induced immune changes were not observed in the *dltX-D-*treated group and 6 out of a total of 30 changes were only observed after treatment with *dltX-D*. Strikingly, not only pro-inflammatory immune responses were reduced in the absence D-Ala substitution, but also anti-inflammatory responses, such as the generation of regulatory T cells. This effect was unexpected, as until now only pro-inflammatory effects were attributed to D-Ala substitution of the teichoic acid backbone [9]–[13]. However, not all *L. plantarum-*induced immunomodulatory effects were influenced by D-Ala substitution of TAs, as 7 out of 22 immune changes were observed after treatment with both the wild-type strain and the *dltX-D* mutant. Both D-Ala dependent and independent immune changes were observed in the intestinal as well as the systemic immune compartment. These results suggest that, in addition to the pro-inflammatory role of *L. plantarum* TA D-Ala *in vitro* [11], [12], [57], this compound is also necessary for the modulation of anti-inflammatory immune responses *in vivo*.

Probiotics are generally marketed as means to prevent disease in healthy individuals. However, studies investigating their mechanism of action have focused mainly on specific (intestinal) disease models [11], [58]. In those models, the intestinal immune barrier is often compromised, altering the contact between the immune cells and the probiotic bacteria [59]. Further, immune homeostasis is often severely compromised to induce disease [58], [60]. Therefore, studies in the disease state hardly reflect and predict the immunomodulatory effects of the bacteria in the healthy intestine, e.g. during immune homeostasis. Knowledge on how different bacterial strains affect the local and systemic immune system in the absence of disease will gain mechanistic insights in bacterial-host interactions and will help clarify the magnitude of their effects in non-diseased individuals. For these reasons, we chose to study the immunomodulatory effects of *L. plantarum* WCFS1 and its TA D-Ala negative derivative in healthy, non-diseased mice.

In the healthy intestine, probiotic bacteria may establish their immunomodulating effects either by direct interactions between host cells and bacterial cell envelope molecules or indirectly by the secretion of metabolites that interact with host cell receptors [3]. Direct interactions between specific molecules on the probiotic cell surface and immune cells has been studied extensively *in vitro*, however, whether these interactions are also established when the intestinal barrier is intact and contribute to the *in vivo* probiotic-induced immune-modulatory effects remains obscure. In this study, we demonstrate that *L. plantarum* D-alanylated TAs in the bacterial wall account for several of the *L plantarum*-induced intestinal and systemic immunomodulatory effects. Although we cannot exclude that small quantities of soluble TA released from lysed *L. plantarum* [61], [62] establish the observed immunomodulatory effects, our data suggest that also when the intestinal barrier is intact, direct interactions between host cells and *L. plantarum* are established and responsible for local and systemic immunomodulation.

To date, the leading dogma is that LTAs initiate pro-inflammatory responses, both *in vitro* and *in vivo* [11], [18]–[22]. We, however, show in healthy animals that also anti-inflammatory responses are influenced by *L. plantarum* LTAs. This suggests that *in vivo*, in healthy mice, the balance between generating pro- and anti-inflammatory responses is differently regulated by LTA than in the disease models. This can be explained by the fact that in the disease models the immune system is already engaged and primed towards a pro-inflammatory response. This might be related to an altered TLR2 signaling [8]. Although TLR2-induced pathways were long viewed as mere pro-inflammatory [63], recent publications demonstrate the induction of anti-inflammatory responses as well [64]–[68]. This was elegantly demonstrated by Manicassamy *et al* [65], who showed that zymosan-induced TLR2 signaling promotes IL10 secretion and the differentiation of regulatory T cell *in vitro*, followed by suppression of experimental autoimmune encephalomyelitis *in vivo* [65]. This anti-inflammatory effect of TLR signaling has been confirmed using several other TLR(2) agonists and in several other experimental inflammation models [64]–[68]. The TLR2 dependent balance between pro- and anti-inflammatory responses may be dependent on the composition of the TLR2 agonist, the amount of TLR2 agonist present as well as the cell type expressing TLR2. The activation of antigen-presenting cells by TLRs is also decisive in many essential processes that lead to the development T cell activation, enhancement of antigen presentation, increased expression of accessory molecules [for example, cluster of differentiation 80 (CD80)], and suppression of regulatory T cells activity [21]. The current view is that TLR signaling in generating *in vivo* immune responses is far more complex than the pro-inflammatory role that was always assumed. This may also explain the differential immunomodulatory properties of LTA in healthy animals as compared to diseased models [21].

One might argue that the absence of effects on the regulatory T cell compartment after treatment with *dltX-D* are the result of a low bacterial load, due to increased bacterial instability [8], [69], rather than altered immunomodulation. However, several arguments support altered immunomodulation rather than this immunological ignorance. First, we showed that although some immunomodulatory effects were abolished after deletion of the *dlt* operon, other immunomodulatory effects were still observed in the same magnitude as observed after *WT-*treatment. Second, even *dltX-D*-treatment specific immunomodulatory effects were observed, both in the intestine as well as in systemic immune compartments. These effects are not expected when the bacterial load drops below the intestinal immunological detection limit. Our results therefore suggest that also our D-Ala negative derivative is able to reach the intestine in adequate numbers and once in there modulates the local and systemic immune system.

Strikingly, we found a different immunomodulatory effect using *dltX-D* than Grangette *et al* in a study using an *L. plantarum dltB* mutant. Grangette *et al* found enhanced protection from TNBS-induced colitis using a *dltB* mutant [11], and suggested that the absence of *L. plantarum* LTA D-alanylation improves its anti-inflammatory capacity *in vivo*. We found that the absence of teichoic acid D-alanylation not only abolished the generation of pro-inflammatory responses, but also the generation of anti-inflammatory responses. How could two bacterial strains, similarly defective in TA D-Ala substitution, perform so similar *in vitro* [8], [11] and yet so different *in vivo*? At this point it is difficult to compare the *in vivo* performance of the two strains as Grangette *et al* measured only a limited numbers of immune parameters in the intestine of diseased mice, whereas we measured the effect on a broad range of immune cells in healthy mice. In the diseased intestine the bacterial-host interactions may be completely different from the interactions in the healthy intestine, as the mucosal barrier has been disrupted to induce disease [70], [71]. This disruption may alter the contact between the bacteria and the intestinal immune cells [72], which may therefore not reflect the bacterial-host interactions that are established in healthy animals or humans [73]. Also the immune response in disease models is often harsh and skewed to s specific T helper response [58], [60], which may therefore not reflect the responses that would be established in healthy animals. Therefore, based on the applied models, the outcome of the two studies is difficult to compare. Further, also differences in the mutant strains exist; Grangette *et al* generated a mutant in which *dltB* was deleted [11], whereas in our study a mutant was employed in which the complete *dltX-D* operon was deleted [8]. Although both mutants are similar with respect to D-Ala substitution of TA, deletion of the *dltB* operon resulted in 3-fold longer LTA in the *L. plantarum* envelope [69], which was not observed after deletion of *dltX-D* [8]. This altered LTA structure may affect bacterial-host interactions and immune outcome *in vivo*. Hence, although in both strains the *dlt* operon was mutated to abolish D-alanylation, the outcome in cell envelope composition appears to differ, which may differently affect immune responses *in vivo.*

Besides the effects of teichoic acid D-Ala substitution on *in vivo* immunomodulation, 9 out of 24 *L. plantarum* WCFS1 induced immune changes were not affected by the absence of TA D-Ala substitution. This demonstrates that besides teichoic acids, *L. plantarum* WCFS1 may have other secreted or membrane-bound effector molecules that contribute to its immunomodulatory properties [6], [74], [75]. The effect of WTA on immunomodulation *in vitro* has been ascribed to shielding effects, rather than direct interactions with immune cell receptors [3], [8]. In our study, the observed *dltX-D* specific immunomodulatory effects may also be the consequence of altered WTA shielding of immunogenic molecules on the *L, plantarum* WCFS1 cell envelope. For example, immunogenic molecules that are normally shielded from interacting with host immune cell receptors, may be available for interactions with the host in the absence of WTA D-Ala substitution. Our results demonstrate that although specific *in vivo* immunomodulatory properties can be attributed to specific bacterial effector molecules, probiotic-induced immunomodulation *in vivo* is a complex and redundant interplay of different host-microbe interactions. However, studies into the role of specific envelope components in probiotic-induced immunomodulation and studies into their interplay will eventually open up possibilities to design probiotic strains with tailored immunomodulatory properties.

In summary, the current study provides insight in host-microbe interactions, by demonstrating the involvement of D-alanylation of bacterial cell envelope components in both intestinal as well as systemic immunomodulation *in vivo*. Even when the intestinal barrier is intact, interaction between immune cells and bacterial envelope components appears indispensable for probiotic-induced immunomodulation *in vivo* [76]. With the acquired knowledge, probiotic-induced health effects could be further exploited by specific modulation of the bacterial envelope composition, for instance by modifications in industrial fermentation media.
